# Inflammation and Colorectal Cancer: A Meta-Analysis of the Prognostic Significance of the Systemic Immune–Inflammation Index (SII) and the Systemic Inflammation Response Index (SIRI)

**DOI:** 10.3390/ijms25158441

**Published:** 2024-08-02

**Authors:** Otilia Menyhart, János Tibor Fekete, Balázs Győrffy

**Affiliations:** 1 Cancer Biomarker Research Group, Institute of Molecular Life Sciences, Hungarian Research Network, 1117 Budapest, Hungary; menyhart.otilia@semmelweis.hu (O.M.); fekete.janos.tibor@semmelweis.hu (J.T.F.); 2 Department of Bioinformatics, Semmelweis University, 1094 Budapest, Hungary; 3 Department of Biophysics, Medical School, University of Pecs, 7624 Pecs, Hungary

**Keywords:** colorectal carcinoma, mortality, overall survival, recurrence-free survival, systematic review

## Abstract

The overall prognosis for colorectal cancer (CRC) remains challenging as the survival time varies widely, even in patients with the same stage of disease. Recent studies suggest prognostic relevance of the novel markers of systemic inflammation, the systemic immune–inflammation index (SII), and the systemic inflammation response index (SIRI). We conducted a comprehensive meta-analysis to assess the prognostic significance of the SII and the SIRI in CRC. We searched the relevant literature for observational studies, and random effects models were employed to conduct a statistical analysis using the metaanalysisonline.com platform. Pooled effect sizes were reported with hazard ratios (HRs) and corresponding 95% confidence intervals (CI). Data from 29 studies published between 2016 and 2024, comprising 10,091 participants, were included in our meta-analysis on SII. CRC patients with high SII levels had worse disease outcomes, which were associated with poor OS (HR: 1.75; 95% CI: 1.4–2.19) and poor PFS/DFS/RFS (HR: 1.25; 95% CI: 1.18–1.33). This increased risk of worse OS was present irrespective of the treatment strategy, sample size (<220 and ≥220), and cutoff used to define high and low SII (<550 and ≥550) groups. Based on data from five studies comprising 2362 participants, we found a strong association between the high SIRI and worse OS (HR: 2.65; 95% CI: 1.6–4.38) and DFS/RFS (HR: 2.04; 95% CI: 1.42–2.93). According to our results, both the SII and SIRI hold great promise as prognostic markers in CRC. Further validations are needed for their age- and stage-specific utility in the clinical routine.

## 1. Introduction

Colorectal cancer (CRC) represents a significant global health burden, ranking as the third most common malignancy. In 2020, CRC accounted for an estimated 935,173 deaths worldwide, making it the second leading cause of cancer-related mortality. The incidence rates of CRC vary considerably across countries with different Human Development Index (HDI) levels. The United States and China report the highest numbers, with steady annual increases, while Japan; Germany; and European countries like the United Kingdom, France, and Italy also show gradual rises. Emerging economies such as India and Brazil are experiencing upward trends due to lifestyle changes and aging populations [1]. Notably, CRC-related deaths are rising in countries with low to medium HDI, whereas countries with high HDI show decreasing trends [2].

A particularly concerning trend is the rising CRC-related mortality among younger populations, with increasing rates observed in individuals under 50 [3]. This increase may be due to earlier exposure to risk factors and the aggressive nature of the disease in younger individuals: patients with early-onset CRC are more frequently diagnosed at advanced stages of the disease (27% compared to 21% in older patients) [4]. By 2040, the burden of CRC is expected to escalate to 3.2 million new cases and 1.6 million deaths, with most cases predicted in highly developed countries [5]. Given the increasing rates, especially in transitioning countries and younger adults, there is an urgent need to understand this highly prevalent disease better.

CRC screening is strongly recommended for average-risk populations between 50 and 75 years of age, although starting at age 45 may offer moderate benefits [6]. CRC tests are either stool-based, such as fecal occult blood testing, fecal immunochemical testing, or stool DNA testing, or based on visual inspections, including colon capsule endoscopy and flexible sigmoidoscopy. Recent advances in artificial intelligence and genetics have led to new diagnostic tests that require further validation [6].

Five-year net survival rates vary significantly by stage at diagnosis, with nearly 90% survival in stage I patients and 70% in stage II compared to just over 10% in those with stage IV metastatic disease. Age also strongly impacts survival, with markedly decreased rates in older patients, regardless of stage [7]. The prognosis is further influenced by various pathological features and genetic and molecular characteristics of CRC, including microsatellite instability, the presence of *KRAS* and *BRAF* mutations, and *CDX2* expression, among others [8]. The overall prognosis for CRC remains challenging, particularly in more advanced stages, as survival times vary widely, even in patients with the same stage of disease. 

The primary treatment for resectable CRC is surgical removal. Chemotherapy or radiotherapy may be used as neoadjuvant or adjuvant treatments before or after surgery to reduce or cure the disease [9,10]. The standard chemotherapy after surgery is fluorouracil (5-FU) combined with leucovorin (LV, folinic acid), a chemoprotectant, to potentiate the activity of 5-FU and prevent its adverse effects. Adjuvant chemotherapy is recommended for all patients with stage III colon cancer, but its benefit for stage II patients remains uncertain despite extensive clinical trials [9]. Non-resectable metastatic CRC may be treated by radiotherapy, cytotoxic chemotherapy, targeted therapy, immunotherapy, and combination therapies [10]. In first-line treatment, 5-FU/LV combined with oxaliplatin (FOLFOX), irinotecan (FOLFIRI), or both (FOLFOXIRI) significantly improves survival in the metastatic setting, and numerous clinical trials are investigating the neoadjuvant efficacy of various approaches, including systemic, immune, and targeted therapies [11,12]. Genomic profiling is crucial, as tailoring treatment to the molecular and pathological features of the tumor significantly improves overall survival (OS). Nevertheless, despite combination therapies, more than half of patients relapse to multidrug-resistant disease [13], and the mortality rate remains relatively high among CRC patients [3].

Assessing molecular features requires costly and sophisticated procedures that are not part of routine laboratory tests. Additional cost-effective, noninvasive, and clinically accessible prognostic markers are needed to tailor treatment and improve patient outcomes.

Besides tumor intrinsic factors, cancer progression is also driven by complex systemic processes [14]. Tumor-promoting inflammation has long been recognized as a hallmark of cancer [15]. Chronic, dysregulated, persistent, and unresolved systemic inflammation plays a crucial role in the development, invasion, metastasis, and drug resistance of cancer [16], particularly in CRC [17]. Several hematological biomarkers have been utilized to assess the systemic inflammatory response, including the levels of neutrophils, platelets, lymphocytes, C-reactive protein, neutrophil-to-lymphocyte ratio (NLR), lymphocyte-to-monocyte ratio (LMR), and the platelet-to-lymphocyte ratio (PLR). Their main advantage is that they can be easily calculated from routine blood tests and do not require expensive equipment and setups. These markers may also be used as prognostic biomarkers to identify high-risk patients who may not be easily detected using traditional clinicopathologic features [18]. For example, NLR and PLR are associated with the size and stage of cancer, making these markers useful for the early diagnosis and prognosis of CRC [19]. 

A more recent blood-based biomarker is the systemic immune–inflammation index (SII), calculated as SII = platelet count × neutrophil count/lymphocyte count. A high SII value indicates a relative increase in neutrophils and platelets and a decrease in lymphocytes, suggesting a pro-tumor inflammatory state and compromised immune surveillance. Since its first application to predict the outcome of hepatocellular carcinoma [20], SII has been associated with the prognosis and clinicopathological characteristics of numerous tumors, including CRC [21]. An earlier meta-analysis confirmed the association between SII and OS [21]; however, since 2020, numerous new data have been accumulated on the prognostic significance of SII on CRC. To achieve a more comprehensive assessment of the prognostic value of SII, we conducted a thorough meta-analysis to assess the significance of SII on OS, disease-free survival (DFS), progression-free survival (PFS), and recurrence-free survival (RFS) in CRC.

Another inflammation-based prognostic marker is the systemic inflammation response index (SIRI), calculated as SIRI = neutrophil count × monocyte count/lymphocyte count. Since its introduction in 2016 as a prognostic factor of pancreatic cancer outcomes [22], the SIRI has received significant attention in various cancers [23]. Although various meta-analyses have assessed the role of SIRI concerning cancer outcomes, to the best of our knowledge, this is the first systematic study to investigate its role in CRC. 

We searched the relevant literature and conducted a comprehensive meta-analysis to assess the prognostic significance of the systemic inflammation-linked indices SII and SIRI in CRC. 

## 2. Methods 

### 2.1. Literature Retrieval

We searched the PubMed database for eligible studies published up to 1 April 2024. The following search phrases were used for literature retrieval on SII: (CRC OR colorectal carcinoma OR colorectal tumor OR colorectal cancer OR colorectal neoplasms OR colonic neoplasms OR colon cancer OR rectal cancer OR rectal cancers OR rectal tumor) AND (systemic immune-inflammatory index OR systemic immune-inflammation index OR SII OR systemic-immune-inflammation index). 

The following search phrases were used for literature retrieval on the SIRI: (CRC OR colorectal carcinoma OR colorectal tumor OR colorectal cancer OR colorectal neoplasms OR colonic neoplasms OR colon cancer OR rectal cancer OR rectal cancers OR rectal tumor) AND (systemic inflammation response index OR SIRI OR systemic-inflammatory response index OR systemic inflammation-response index). Since data from published studies were used, no ethical approval or patient consent was necessary.

### 2.2. Inclusion Criteria

Only full-text English studies were included, containing the following details: hazard rates (HR) and the corresponding 95% confidence intervals (95% CI); pretreatment SII or pretreatment SIRI; outcomes, i.e., overall survival (OS), recurrence-free survival (RFS), progression-free survival (PFS), or disease-free survival (DFS); and pretreatment SII or SIRI cutoff-values. We excluded papers with insufficient information, conference abstracts, letters, editorials, reviews, case reports, animal studies, and studies focusing on basic research.

### 2.3. Data Extraction

One investigator, OM, extracted the data. Questionable results were resolved by discussing with JTF and BGy. The following pieces of information were extracted: name of the first author, publication year, country of origin, cancer type (CRC or colon or rectal carcinoma), study period, study design, sample size, age of patients (median and range where available; otherwise, the number of patients older than 60 years of age), sex distribution, TNM stage, treatment type, the cutoff value for SII, the cutoff value for SIRI, methods for the cutoff value selection, follow-up period, survival endpoints, and the corresponding HR values with 95% CI values. The TNM staging system is a widely used cancer classification framework that describes the extent of cancer spread: T (Tumor) refers to the size and extent of the primary tumor, N (Nodes) indicates whether and how much the cancer has spread to nearby lymph nodes, and M (Metastasis) describes whether the cancer has spread to other parts of the body. This system helps guide treatment decisions and predict patient outcomes.

### 2.4. Statistical Analysis

OS and PFS/DFS/RFS were analyzed to calculate the prognostic effects of SII on CRC outcome, reported as pooled HR and 95% CI values. Similar outcome measures for PFS/DFS/RFS were integrated for the ultimate analysis, but were also assessed separately. OS and the integrated PFS/DFS were analyzed to calculate the prognostic effects of SIRI on the CRC outcome, which were reported as HR and 95% CI values. Heterogeneity was assessed using the chi-squared (χ^2^)-based Q statistic and the inconsistency index (I^2^) [24]. Statistically significant heterogeneity was defined by a χ^2^ *p*-value of less than 0.1 or an I^2^ value greater than 50%. A random-effects model was applied for the analysis in cases of significant heterogeneity. Potential publication bias was examined using the Egger’s tests. 

Subgroup analyses were performed based on sample size, tumor location, TNM stage, treatment, cutoff value, cutoff selection method, and country of the investigation. Sensitivity analysis was also conducted to evaluate the effect of the individual study data on the HRs of OS and PFS/DFS/RFS. All statistical analyses were performed using metaanalysisonline.com, a free online statistical software (https://metaanalysisonline.com, accessed on 5 May 2024). 

## 3. Results

### 3.1. Studies Investigating the Prognostic Significance of SII in CRC

**Figure 1:**
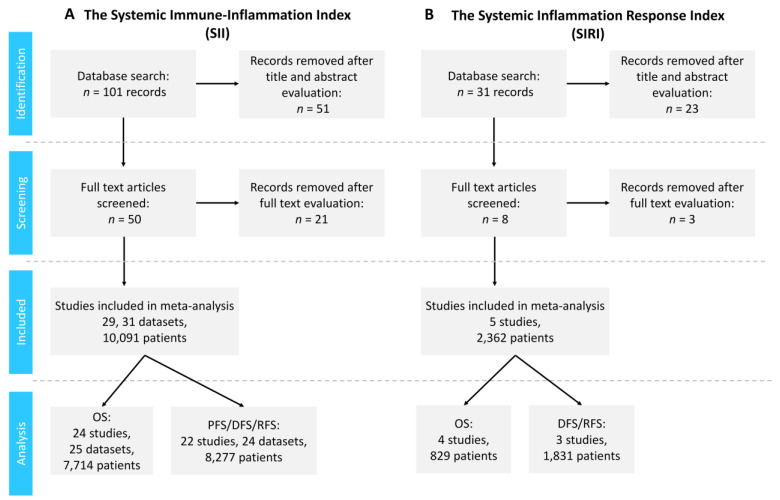
Flowchart of the study selection process. Selection of studies for the systemic immune–inflammation index (SII, (**A**)) and the systemic inflammation response index (SIRI, (**B**)).

According to the retrieval strategy, a total of 101 studies related to the prognostic significance of SII in CRC were identified. Upon reviewing the titles and abstracts, 50 articles remained. After reading the full text, additional studies were removed due to missing essential information (mostly lacking the univariate HR and/or 95% CI values). The authors of one study were contacted for the necessary univariate HR values not included in the original publication, and they provided us with the data [25]. Ultimately, 29 studies containing 31 datasets, published between 2016 and 2024 and comprising 10,091 participants, were included in our meta-analysis [25,26,27,28,29,30,31,32,33,34,35,36,37,38,39,40,41,42,43,44,45,46,47,48,49,50,51,52,53] (Figure 1A). Of the identified studies, 27 were retrospective and 2 [32,33] were prospective trials. Twenty-two studies were conducted in China, three in Italy [27,32,33], two in Japan [34,44], and one in Hungary [25] and the USA [47], respectively, and the number of participants ranged between 41 and 1383. Twenty-two datasets investigated CRC, two datasets colon cancer [36,50], five datasets rectal cancers [27,29,35,48,51], and one colon cancer- or rectal cancer-related liver-only metastases (CLM or RLM, respectively) [25]. The main characteristics of the included studies are illustrated in Table 1.

### 3.2. Studies Investigating the Prognostic Significance of SIRI in CRC

Based on a PubMed search, we identified 31 studies. Upon reviewing the titles and abstracts, we discarded 23 studies, mainly due to irrelevant information. After reviewing the remaining full-text articles, we discarded three additional studies due to missing information. Ultimately, five studies published between 2016 and 2024, comprising 2362 participants, were identified and included in our meta-analysis (Figure 1B) [46,54,55,56,57]. All identified studies were retrospective. Four studies were conducted in China and one in Turkey, and the number of participants ranged between 104 and 1014. Four studies investigated CRC, and one studied rectal cancer. The characteristics of the included studies are listed in Table 2.

### 3.3. Prognostic Impact of SII on OS in CRC Patients

**Figure 2:**
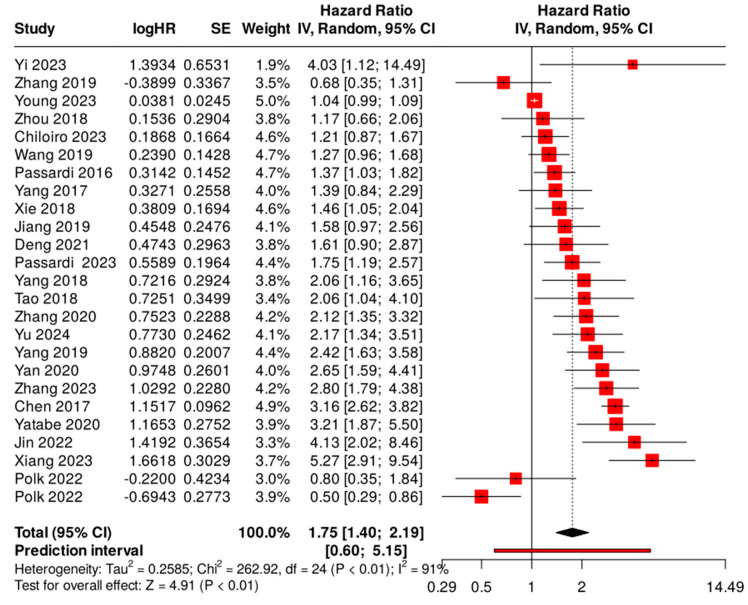
Forest plot of the association between the systemic immune–inflammation index (SII) and overall survival (OS) in patients with CRC. Each red square represents the point estimate of the HR for a study, with the size of the square proportional to the study’s weight in the overall analysis. The horizontal lines through the squares represent the 95% confidence intervals (CI). The black diamond at the bottom of the plot represents the combined HR from the meta-analysis, with the width of the diamond representing the 95% CI. The dotted vertical line represents a HR of 1.0, indicating no effect. Studies included: [25,26,27,28,30,31,32,33,36,37,38,39,40,41,42,43,44,45,47,48,49,51,52,53].

Twenty-five datasets from 24 studies containing 7714 patients provided data for the OS analysis. A random effect model with an inverse variance method was performed due to significant heterogeneity detected in the data (I^2^ = 90.9%, *p* < 0.01). The pooled HR from the included studies was 1.75 (95% CI: 1.4–2.19), indicating a significant association between high SII value and poor OS in CRC (see Figure 2 and Table 3). The test for overall effect was significant at *p* < 0.05. 

Subgroup analysis was conducted based on the sample size, tumor location, tumor stage, treatment procedure, cutoff value of SII, cutoff selection method, and country of investigation. High SII was consistently linked to worse OS, except for one study containing two datasets from Hungary and one from the USA (Table 3). Moreover, the relationship between high SII and worse outcomes was not significant in stage IV tumors, probably due to the already very poor outlook for these patients. Nevertheless, high SII predicted poor OS in Chinese, Italian, and Japanese patients, and high SII was linked to worse outcomes irrespective of treatment, SII cutoff value, the method of SII cutoff selection, the study’s sample size, and the type of cancer in the pooled analysis involving all patients (Table 3).

### 3.4. Prognostic Role of SII for PFS/DFS/RFS

**Figure 3:**
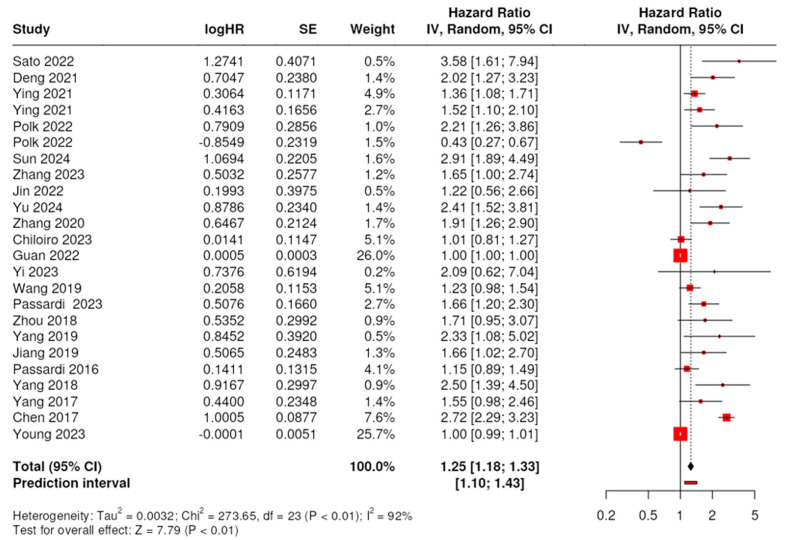
Forest plot of the association between the systemic immune–inflammation index (SII) and the integrated survival outcome, PFS/DFS/RFS, in patients with CRC. Each red square represents the point estimate of the HR for a study, with the size of the square proportional to the study’s weight in the overall analysis. The horizontal lines through the squares represent the 95% confidence intervals (CI). The black diamond at the bottom of the plot represents the combined HR from the meta-analysis, with the width of the diamond representing the 95% CI. The dotted vertical line represents a HR of 1.0, indicating no effect. Studies included: [25,26,27,28,29,30,31,32,33,34,35,37,41,42,43,45,46,47,48,50,51,53].

Twenty-four datasets from 22 publications covering 8277 patients were included in the integrated PFS/DFS/RFS outcome analysis. Due to significant heterogeneity in the data (I^2^ = 91.6%, *p* < 0.01), a random effect model was performed to study the association between the outcome measures and SII values. The analysis showed a significant association between high SII and worse disease outcomes, with a pooled HR of 1.25 (95% CI: 1.18–1.33). The test for overall effect was significant at *p* < 0.05 (Figure 3). 

We also investigated each outcome measure individually. Data for the PFS analysis were included from eleven studies covering 3453 patients. Based on the random effects model analysis, the pooled HR was 1.64 (95% CI: 1.2–2.24); thus, elevated SII was linked to a worse PFS among CRC patients. 

Data for DFS analysis were extracted from seven studies covering 2752 patients. By performing a random effects model, we identified an HR of 1.56 (95% CI: 1.14–2.16); thus, the elevated SII was linked to a worse DFS in CRC patients. Data for RFS analysis were included from four studies covering 2072 patients. The pooled HR was 1.48 (95% CI: 0.94–2.35), and the association between high SII and poor DFS was not significant. 

According to the subgroup analysis, high SII was associated with poor PFS/DFS/RFS irrespective of the study’s sample size, the diagnosis of CRC or rectal cancer, the cutoff value, and the cutoff selection method. We found that geographic variation across studies affected the association (Table 4); moreover, the link between high SII and worse disease outcomes was not significant in stage IV patients.

### 3.5. Prognostic Impact of SIRI on OS in CRC Patients

**Figure 4:**
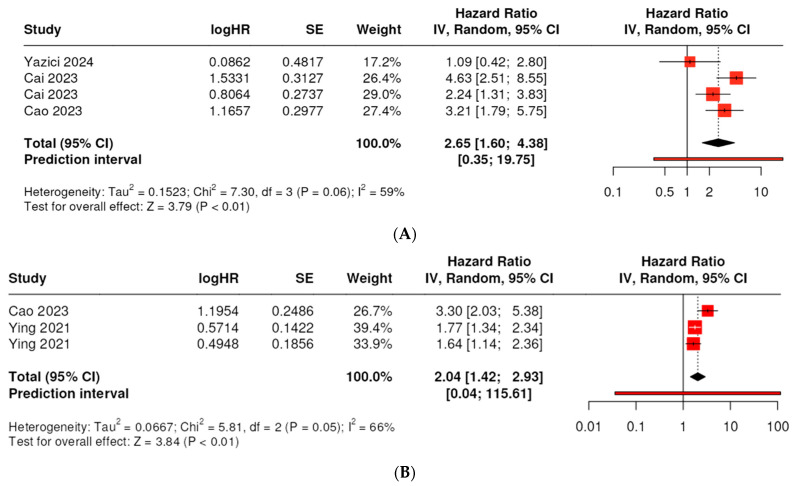
Forest plot of the association between the systemic inflammation response index (SIRI) and overall survival (OS); studies included: [54,55,56,57] (**A**), and the SIRI and the integrated outcome measure DFS/RFS in patients with CRC; studies included [46,57] (**B**). Each red square represents the point estimate of the HR for a study, with the size of the square proportional to the study’s weight in the overall analysis. The horizontal lines through the squares represent the 95% confidence intervals (CI). The black diamond at the bottom of the plot represents the combined HR from the meta-analysis, with the width of the diamond representing the 95% CI. The dotted vertical line represents a HR of 1.0, indicating no effect.

Four studies involving 829 patients investigated the prognostic role of SIRI on the OS of CRC patients. We observed significant heterogeneity in the data (I^2^ = 59%, *p* = 0.06). By using a random effects model, we identified a pooled HR of 2.65 (95% CI: 1.6–4.38) across the eligible studies; thus, the elevated SIRI was linked to a significantly worse OS in CRC (Figure 4A). The test for overall effect was significant at *p* < 0.05.

### 3.6. Prognostic Impact of SIRI on DFS/RFS in CRC Patients

Three datasets from two publications, covering 1831 patients, were included in the integrated DFS/RFS outcome analysis. There was significant heterogeneity detected in the data (I^2^ = 66%, *p* = 0.05). We conducted the analysis using a random effects model, which showed a significant association between high SIRI and worse disease outcomes, with a pooled HR of 2.04 (95% CI: 1.42–2.93) (Figure 4B). The test for overall effect was significant at *p* < 0.01.

### 3.7. Sensitivity Analysis for the Association between SII or SIRI and the Outcome Measures

We conducted a sensitivity analysis by sequentially omitting each study from the pooled HR analysis. The association between high SII and worse OS or high SII and worse PFS/DFS/RFS did not change by leaving out any one study from the analysis, supporting the reliability of our results. The association also remained significant when single studies involving SIRI, OS, SIRI, and DFS/RFS were omitted from the analysis. 

### 3.8. Publication Bias

#### 3.8.1. The Association between SII and Disease Outcome

**Figure 5:**
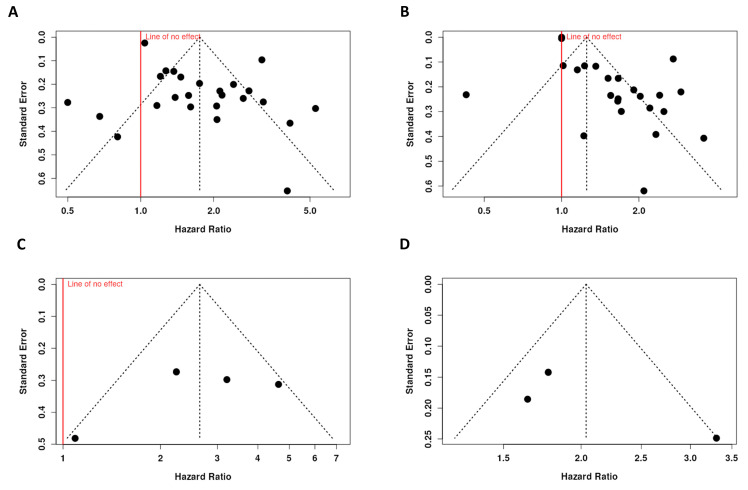
Publication bias was tested using Egger’s test for the association between SII and overall survival (**A**); SII and PFS/DFS/RFS (**B**); SIRI and overall survival (**C**); and SIRI and DFS/RFS (**D**). In each plot, the black dots represent individual studies included in the meta-analysis. The vertical dotted line indicates the combined hazard ratio (HR) from the meta-analysis, while the diagonal dotted lines represent the expected distribution of studies in the absence of publication bias (the “funnel” shape). The solid red line indicates the line of no effect (HR = 1.0).

We assessed publication bias in the included studies to investigate the association between SII and OS with an Egger’s test. The funnel plot asymmetry indicated a potential publication bias among the included studies (intercept: 2.44, 95% CI: 1.07–3.8, t: 3.506, *p* = 0.002). Similarly, in the studies linking the SII and PFS/DFS/RFS, the asymmetry in the funnel plot indicated a potential publication bias (intercept: 2.35, 95% CI: 1.29–3.41, t: 4.342, *p* < 0.001) (Figure 5A,B).

#### 3.8.2. The Association between SIRI and Disease Outcome

The funnel plot did not indicate a potential publication bias in the studies investigating the association between SIRI and OS. The Egger’s test did not support the presence of funnel plot asymmetry (intercept: −4.07, 95% CI: −12.09–3.95, t: −0.995, *p* = 0.425). Additionally, there was no publication bias among the studies investigating the association between SIRI and DFS/RFS; the Egger’s test did not support the presence of funnel plot asymmetry (intercept: 5.07, 95% CI: −2.44–12.58, t: 1.323, *p* = 0.412) (Figure 5C,D).

## 4. Discussion

We examined the association between two markers of systemic inflammation in peripheral blood and various outcome measures in CRC patients. A previous meta-analysis published in 2020 summarized eleven studies exploring the association between SII and OS and eight studies investigating the link between SII and PFS in CRC patients [21]. However, numerous additional studies have explored this relationship over the past four years. We collected data from 29 studies involving 10,091 patients to clarify the role of SII in the prognosis of CRC. Our findings indicate that high systemic inflammation, as measured by the SII, is a strong marker of poor disease outcomes and is closely associated with worse overall survival. Elevated SII was also linked to poorer PFS/DFS/RFS outcomes. 

We also investigated the association between SIRI and CRC prognosis. SIRI was initially developed to predict survival outcomes in patients with advanced pancreatic cancer undergoing chemotherapy and has effectively reflected the systemic inflammation status [22]. Due to its accessibility, the prognostic value of SIRI has been studied across various cancers, including malignancies of the urinary, respiratory, and digestive systems and head and neck cancers [23]. A meta-analysis of 30 studies and 10,754 cases found a strong association between high SIRI, low OS, and low DFS/RFS/PFS across all investigated cancers [58]. However, previous meta-analyses did not include studies on CRC. We found a significant association between high SIRI and worse OS or DFS/RFS in CRC patients, making our study the first to address this gap. The extended follow-up period, ranging between 24 and 90 months (even up to 96 months in some studies), reflects the long-term survival impacts of the pretreatment systemic inflammation.

Although most studies supported the associations between high SII and SIRI and poor disease outcomes, several studies presented inconsistent results. For instance, the study by Polk et al. [25] reported no association between high SII and worse OS or DFS in patients with colon- or rectal-cancer-associated liver metastases. This discrepancy could be due to the specific patient population, focusing solely on those with liver metastases, who may respond differently to systemic inflammation markers compared to those with primary CRC. Similarly, the study by Young et al. [47] found no significant association between high SII and OS, potentially influenced by the small sample size (41 patients) and the unique treatment modality (transarterial radioembolization), which could affect the systemic inflammatory response differently than conventional treatments. Finally, Yan et al. [40] reported a non-significant association between high SII and poor OS. The patient subgroup with synchronous peritoneal carcinomatosis may have had distinct inflammatory responses or treatment regimens that impacted the prognostic value of SII differently. These inconsistent findings highlight the importance of considering patient heterogeneity and disease subtypes and underscore the potential influence of specific metastatic sites, treatment modalities, and patient demographics on the prognostic value of systemic inflammation markers. Future studies are needed to validate the prognostic value of SII and SIRI in diverse CRC subpopulations and to explore the underlying mechanisms driving these associations.

The SII and SIRI capture different aspects of systemic inflammation. The SII reflects the balance between pro-tumor inflammatory cells, such as neutrophils and platelets, and anti-tumor immune cells, such as lymphocytes [20]. High SII values indicate a higher inflammatory state, often associated with poor prognosis in cancer patients [59]. Neutrophils, or polymorphonuclear leukocytes, are among the most active cells of the innate immune system and possess several pro-oncogenic properties. They contribute to cancer progression by promoting angiogenesis, inducing immunosuppression, and facilitating metastasis [60]. By releasing cytokines and growth factors, neutrophils can foster an inflammatory environment that supports cancer cell proliferation and spread [61]. The direct and indirect interaction between platelets and tumor cells also helps to sustain tumor progression and metastasis, immune escape, and chemoresistance [62]. For instance, platelets can adhere to the surfaces of tumor cells, forming microaggregates that create a physical shield, protecting the tumor cells from attacks by immune cells [63]. Moreover, platelets facilitate the arrest of cancer cells at the endothelium and their extravasation into distant organs [64]. Thus, elevated platelet counts, reflected in a high SII, could indicate enhanced metastatic potential. 

On the other hand, lymphocytes make up one of the most crucial effector mechanisms in the immunity to cancer. A high peripheral lymphocyte percentage prior to treatment has been reported to be an independent favorable prognostic factor in various tumors, including CRC [65,66]. Moreover, tumor-infiltrating lymphocytes play a significant role in the tumor immune environment [67]. A meta-analysis of 43 trials involving 21,015 CRC patients showed that high levels of tumor-infiltrating lymphocytes were associated with improved OS and DFS [68].

Compared to SII, the SIRI highlights the role of monocytes, neutrophils, and lymphocytes in the inflammatory response. Monocytes are precursors to macrophages and dendritic cells involved in tumor-associated inflammation and immune regulation. The peripheral monocyte count is closely associated with the density of tumor-associated macrophages (TAMs), creating a microenvironment favorable for cancer development that is linked to poor prognosis [69]. Consequently, the peripheral monocyte count serves as a valuable prognostic marker that reflects the status of the tumor microenvironment; thus, SIRI provides additional insights into the interplay between innate and adaptive immunity in the tumor microenvironment. Elevated SII or SIRI due to lymphocytopenia and/or high neutrophil/platelet/monocyte counts indicate decreased immune response against tumor or increased tumor spread or recurrence ability. Nevertheless, the biological mechanism explaining the close correlation between systemic inflammatory indices and poor disease prognosis is still unclear and requires additional research.

Forecasting prognosis in CRC is increasingly challenging, particularly in advanced disease stages and elderly patients, which reinforces the need for innovative markers to guide treatment decisions. The clinical implications of SII and the SIRI as prognostic markers in CRC are valuable. These indices can be routinely measured as part of the standard diagnostic workup, using data from complete blood counts to facilitate risk stratification and treatment planning. Our meta-analysis suggests the usefulness of SII across diverse demographics, including the younger, early-onset patients [38]. Thus, patients with a high SII or SIRI might benefit from more aggressive systemic therapies or closer surveillance post-surgery.

Nevertheless, translating SII and SIRI into personalized prognoses and treatment decisions for individual patients is challenging because the cutoff values for classifying high-risk and low-risk patients may vary across different ages and stages of the disease. Moreover, neutrophil and monocyte counts can differ significantly between individuals. One strategy to maximize the utility of SII and SIRI would be longitudinal surveillance of patients, performed over time with regular analyses. Increasing SII or SIRI values over time may signal disease recurrence or progression. Combining these indices with other biomarkers could further enhance personalized treatment strategies, ultimately improving long-term prognostic assessments.

Despite the promising results, our study has several limitations: almost all included studies were retrospective in design, with a limited sample size. The significant heterogeneity among the included studies in terms of study design, patient populations, treatment regimens, and follow-up periods may have affected the generalizability of the findings. The SII and SIRI cutoff values were determined using different procedures across studies, which may have impacted the comparability of results. Standardized cutoff values should inevitably be established for broader clinical applications. We extracted the HR values from univariate analyses that may eventually overestimate the effect size. Finally, a significant publication bias was present among the collected studies regarding the SII, where studies with positive findings are more likely to be published than those with negative results, skewing the overall conclusions. In the future, large-scale prospective studies will be required in order to clarify the nature of genuine associations, develop standardized cutoff values, and explore the biological mechanisms underlying the association between high SII/SIRI and poor CRC outcomes. 

In conclusion, understanding the role of systemic inflammation in CRC progression is crucial in order to stratify patients for more personalized therapeutic strategies. It is essential to consider the heterogeneity of CRC and the complex interplay between the immune system, tumor biology, and the tumor microenvironment. According to the results of our meta-analysis, both SII and SIRI hold great promise as prognostic markers in CRC, but require further validations for their age- or stage-specific utility in the clinical routine.

## Figures and Tables

**Table 1:** The main features of the studies included the analysis of SII and outcome measures in CRC patients. The “Histology” column specifies whether the study focuses on colorectal cancer (CRC), colon cancer, rectal cancer, or liver metastases specific to colon or rectal cancer (CLM or RLM, respectively). The column “Study design” refers to the prospective (prospect) or retrospective (retrospect) nature of the study.

First Author	Year	Country	Histology	Study Period	Study Design	Sample Size	Age (Median)	Sex M/F	TNM Stage	Treatment	SII Cutoff	Method for Cutoff Selection	Follow-Up (Months)	Survival Endpoint
Passardi [32]	2016	Italy	CRC	2007–2012	prospect	289	65.5	174/115	I–IV	chemo + targeted therapy	730	X-tile	36 (1–65)	OS PFS
Chen [26]	2017	China	CRC	1994–2010	retrospect	1383	NA	788/595	I–IV	surgical resection	340	ROC analysis	NA	OS PFS
Yang [41]	2017	China	CRC	2009–2015	retrospect	95	57	58/37	IV	chemo + targeted therapy	460.7	median	40 (12–72)	OS PFS
Xie [39]	2018	China	CRC	2009–2014	retrospect	240	59 (18–90)	157/83	IV	surgical resection	649.5	median	26.7 (1.1–92.4)	OS
Tao [36]	2018	China	colon	2011–2013	retrospect	118	60	63/55	I–IV	surgical resection	667.8	median	36	OS
Yang [42]	2018	China	CRC	2010–2015	retrospect	98	53 (26–83)	59/39	I–IV	neoadjuvant chemo-radiotherapy	437.7	median	37 (16.2–93.3)	OS PFS
Zhou [53]	2018	China	CRC	2007–2015	retrospect	516	16–87	331/185	I–IV	surgical resection	568.7	ROC analysis	21.7 (2.1–118.7)	OS PFS
Wang [37]	2019	China	CRC	2002–2016	retrospect	452	57	289/163	IV	surgical resection	517	X-tile	28	OS PFS
Yang [43]	2019	China	CRC	2009–2015	retrospect	220	57	133/87	III–IV	adjuvant chemo-radiotherapy	534.9	ROC analysis	23.9 (12–87)	OS PFS
Zhang [52]	2019	China	CRC	2010–2013	retrospect	224	67 (30–89)	127/97	I–IV	surgical resection	642.2	median	48	OS
Jiang [30]	2020	China	CRC	2010–2017	retrospect	102	28–75	72/30	IV	chemo + targeted therapy	660.6	ROC analysis	33.2 (2.6–94.5)	OS PFS
Yan [40]	2020	China	CRC	1997–2013	retrospect	103	47 over 60	67/46	III–IV	surgery + chemotherapy	410	ROC analysis	55.4	OS
Yatabe [44]	2020	Japan	CRC	2010–2014	retrospect	733	66 (58–74)	463/270	I–IV	surgical resection	median 550	SII trichotomized into tertiles	36 60	OS
Deng [28]	2021	China	CRC	2006–2016	retrospect	283	57 (25–82)	187/96	I–IV	surgery + chemotherapy	0.0135	ROC analysis	35.4	OS RFS
Ying [46]	2021	China	CRC	2013–2016	retrospect	1014	460 over 60	622/392	II–III	surgery + chemotherapy	665	X-tile	36	RFS
Ying [46]	2021	China	CRC	2013–2016	retrospect	519	328 over 60	622/392	II–III	surgery + chemotherapy	665	X-tile	36	RFS
Guan [29]	2022	China	rectal	2016–2019	retrospect	278	53.97 ± 10.11	181/97	II–IV	neoadjuvant chemo-radiotherapy	540	X-tile	Last follow-up: 31 December 2021	OS DFS
Jin [31]	2022	China	CRC	2012–2015	retrospect	476	60.8 (25–90)	259/217	I	surgical resection	540.3	ROC analysis	68	OS DFS
Polk [25]	2022	Hungary	CRC colon (CLM)	2001–2018	retrospect	67	65	36/31	IV	surgery + chemotherapy	535/290 RFS/OS	ROC analysis	46.5	OS RFS
Polk [25]	2022	Hungary	CRC renal (RLM)	2001–2018	retrospect	103	62	69/34	IV	surgery + chemotherapy	792/742 RFS/OS	ROC analysis	59.8	OS RFS
Passardi [33]	2023	Italy	CRC	2016–2019	prospect	182	33–83	72/60	I–IV	chemo + targeted therapy	730	ROC analysis	52.6	OS PFS
Chiloiro [27]	2023	Italy	rectal	2002–2019	retrospect	808	64 (26–88)	493/315	I–IV	neoadjuvant chemo-radiotherapy	500	log-rank test	53.5 (range 6–198)	OS DFS
Sato [34]	2023	Japan	CRC	2013–2020	retrospect	86	71 (37–93)	50/36	II–IV	surgical resection	597	ROC analysis	35	RFS
Xiang [38]	2023	China	CRC	2013–2017	retrospect	236	45	143/93	I–III	neoadjuvant chemo-radiotherapy, adjuvant therapy	637.6	survminer R package	48	OS
Yi [45]	2023	China	CRC	2017–2021	retrospect	75	47 (23–84)	48/27	IV	chemo+ immuno-therapy	409.6	ROC analysis	24	OS PFS
Young [47]	2023	USA	CRC	2014–2019	retrospect	41	61.4 ± 8.2	21/20	IV	transarterial radio-embolization (TARE), chemotherapy	591.7	median	12	OS PFS
Zhang [50]	2023	China	colon	2013–2018	retrospect	188	67 (33–92)	117/71	I–IV	surgical resection	514.1	median	43	DFS
Zhang [49]	2023	China	CRC	2019–2023	retrospect	160	64 (38–85)	98/62	I–IV	surgical resection	513.5	ROC analysis	29.25 (2–60)	OS

**Table 2:** The main features of the studies that participated in the analysis of SIRI and disease outcomes, OS, and DFS/RFS were as follows. The column “Study design” refers to the prospective (prospect) or retrospective (retrospect) nature of the study.

First Author	Year	Country	Histology	Study Period	Study Design	Sample Size	Age (Median)	Sex M/F	TNMStage	Treatment	SIRI Cutoff	Method for Cutoff Selection	Follow-Up (Months)	Survival Endpoint
Ying [46]	2021	China	CRC	2013–2016	retrospect	1014	460 over 60	622/392	II–III	surgery + chemotherapy	1.95	X-tile	36	RFS
Ying [46]	2021	China	CRC	2013–2016	retrospect	519	328 over 60	348/171	II–III	surgery + chemotherapy	1.95	X-tile	36	RFS
Cao [57]	2023	China	CRC	2013–2017	retrospect	298	56.25	172/126	I–IV	surgical resection	1.4	X-tile	24	OS, DFS
Cai [55]	2023	China	CRC	2015–2017	retrospect	210	121 over/or 60	118/92	I–III	surgery + chemotherapy	2	X-tile	90	OS
Cai [56]	2023	China	CRC	2015–2017	retrospect	217	94 over/or 60	124/93	I–III	surgery + chemotherapy	1.1	X-tile	90	OS
Yazici [54]	2024	Turkey	rectal	2017–2022	retrospect	104	62 (31–89)	59/45	I–IV	surgery + chemotherapy	1.38	ROC analysis	33 (1–62)	OS

**Table 3:** Subgroup analysis of pooled HRs and 95% CIs between SII and OS.

Variables	No. of Datasets	No. of Patients	Effect Model	HR (95%CI)	*p*	Heterogeneity, I^2^%	Heterogeneity, *p*
Total number of datasets	25	7714	random	1.75 (1.4–2.19)	<0.01	90.9	<0.01
Sample size							
<220	11	1144	random	1.55 (1.13–2.13)	<0.01	83.8	<0.01
≥220	14	6570	random	1.9 (1.46–2.49)	<0.01	84.6	<0.01
Tumor location							
CRC	19	5908	random	1.92 (1.47–2.50)	<0.01	92.4	<0.01
Rectal	3	1518	random	1.72 (1.14–2.6)	<0.01	66	0.05
Colon	1	118	na	2.07 (1.04–4.1)		na	na
TNM stage							
I–II or I–III	2	712	random	4.77 (3.02–7.54)	<0.01	0	0.61
I–IV	12	5032	random	1.8 (1.36–2.38)	<0.01	81	<0.01
III–IV	3	390	random	1.91 (1.08–3.38)	0.03	68.7	0.04
IV	7	1108	random	1.2 (0.94–1.53)	0.14	72	<0.01
Treatment							
Neoadjuvant/adjuvant chemoradiotherapy	2	318	random	2.29 (1.66–3.17)	<0.01	0	0.65
Chemotherapy + targeted therapy	4	668	random	1.49 (1.23–1.8)	<0.01	0	0.77
Chemotherapy + immunotherapy	1	75	na	4.048 (1.12–14.49)		na	na
Surgery + chemotherapy	9	2877	random	1.63 (1.02–2.58)	<0.01	89.5	<0.01
Surgical resection	6	2455	random	2.13 (1.39–3.27)	<0.01	76.9	<0.01
Neoadjuvant chemoradiotherapy + surgery	2	1280	random	1.56 (0.9–2.72)	0.11	75	0.05
Cutoff value of SII							
<550	13	4458	random	2.01 (1.54–2.62)	<0.01	79.2	<0.01
≥550	12	3256	random	1.51 (1.15–2.00)	<0.01	86.5	<0.01
Cutoff selection method							
X-tile software	3	1213	random	1.47 (1.13–1.9)	<0.01	47	0.15
ROC analysis	10	3500	random	2.27 (1.78–2.89)	<0.01	63.5	<0.01
Median value	6	1013	random	1.55 (1.15–2.08)	<0.01	49	0.08
Country							
China	18	5491	random	2.2 (1.62–2.53)	<0.01	76.3	<0.01
Italy	3	1279	random	1.39 (1.15–1.69)	<0.01	5	0.35
Japan	1	733	na	3.21 (1.87–5.5)		na	na
Hungary	2	170	random	0.58 (0.37–0.91)	0.02	0	0.35
USA	1	41	na	1 (0.99–1.09)		na	na

**Table 4:** Subgroup analysis of pooled HRs and 95% CIs between SII and PFS/DFS/RFS.

Variables	No. of Datasets	No. of Patients	Effect Model	HR (95%CI)	*p*	Heterogeneity, I^2^%	Heterogeneity, *p*
Total number of datasets	24	8277	random	1.25 (1.18–1.33)	<0.01	91.6	<0.01
Sample size							
<220	10	1037	random	1.52 (1.1–2.1)	<0.01	85.6	<0.01
≥220	14	7240	random	1.61 (1.26–2.06)	<0.01	93.8	<0.01
Tumor location							
CRC	18	6001	random	1.56 (1.23–1.97)	<0.01	92.3	<0.01
Rectal	5	2088	random	1.6 (1.1–2.34)	0.01	91.5	<0.01
Colon	1	188	na	1.65 (0.998–2.7)		na	na
TNM stage							
I or I–III or II–III	4	2301	random	1.67 (1.18–2.36)	<0.01	69.5	0.02
I–IV	9	3985	random	1.76 (1.29–2.39)	<0.01	87.1	<0.01
II–IV or III–IV	5	1123	random	1.91 (1.12–3.25)	0.02	87.2	<0.01
IV	6	868	random	1.09 (0.82–1.45)	0.54	80.7	<0.01
Treatment							
Neoadjuvant/adjuvant chemoradiotherapy	6	2114	random	1.58 (1.14–2.19)	<0.01	86.6	<0.01
Chemotherapy + targeted therapy	4	668	random	1.42 (1.16–1.75)	<0.01	24	0.27
Chemotherapy + immunotherapy	1	75	na	2.09 (0.62–7.04)		na	na
Surgery + chemotherapy	5	1986	random	1.31 (0.82–2.09)	<0.01	89.5	<0.01
Surgical resection	7	3393	random	1.97 (1.37–2.84)	<0.01	83.9	<0.01
Cutoff value of SII							
<550	14	4953	random	1.77 (1.33–2.37)	<0.01	93.8	<0.01
≥550	10	3324	random	1.34 (1.06–1.68)	0.01	85.8	<0.01
Cutoff selection							
X-tile software	6	3024	random	1.28 (1.05–1.55)	0.01	81.3	<0.01
ROC analysis	12	3785	random	1.82 (1.3–2.53)	<0.01	83.2	<0.01
Median value	4	619	random	1.95 (1.52–2.5)	<0.01	0	0.42
Country							
China	17	6701	random	1.76 (1.37–2.28)	<0.01	93.1	<0.01
Italy	3	1279	random	1.22 (0.93–1.59)	0.14	67	0.05

## Data Availability

The original contributions presented in the study are included in the article; further inquiries can be directed to the corresponding author.
